# Establishment and behavioural characterization of a novel constitutive P2X7 receptor knockout mouse line

**DOI:** 10.1007/s11302-025-10074-x

**Published:** 2025-03-01

**Authors:** Iven-Alex von Mücke-Heim, Judit Oldekamp, Michael W. Metzger, Sarah Kläffgen, Hao Tang, Sandra M. Walser, Nina Dedic, Gerhard Rammes, Florian Holsboer, Wolfgang Wurst, Jan M. Deussing

**Affiliations:** 1 Molecular Neurogenetics, Max Planck Institute of Psychiatry, 80804 Munich, Germany; 2 Max Planck Institute of Psychiatry, Research Clinic, 80804 Munich, Germany; 3 Department of Anesthesiology and Intensive Care, Klinikum Rechts Der Isar, 81675 Munich, Germany; 4 Max Planck Institute of Psychiatry, Present Address: HMNC Brain Health, 80539 Munich, Germany; 5 Institute of Developmental Genetics, Helmholtz Zentrum München, 85764 Neuherberg, Germany; 6 Chair of Developmental Genetics, Munich School of Life Sciences Weihenstephan, Technical University of Munich, 85354 Freising, Germany; 7 Munich Cluster of Systems Neurology (SyNergy), Munich, Germany; 8 German Center for Neurodegenerative Diseases (DZNE) Site Munich, 81377 Munich, Germany

**Keywords:** P2X7 receptor, P2rx7 gene, Purinergic, Knockout mice, Behaviour

## Abstract

The P2X7 receptor is an adenosine triphosphate (ATP)-gated ion channel expressed in different cell types of the brain. Polymorphisms in the *P2RX7* gene have repeatedly been associated with psychiatric disorders including major depression. Depression is a stress-related disorder in which a dysregulation of the immune system has attracted increasing attention as a potential disease mechanism. The well-documented role of P2X7 in inflammatory conditions advocates its involvement in immune system dysregulation and depression genesis. However, understanding its exact role requires further research using appropriate animal models. Unfortunately, some of the most widely used P2X7 knockout mouse models are limited in their utility by the continuous expression of certain *P2rx7* splice variants or even activation of de novo transcripts. To overcome this limitation, we generated a novel constitutive and complete P2X7 KO mouse line. These KO mice lack all known murine splice variants and protein expression resulting in a loss-of-function as confirmed by calcium imaging and by the inability of P2X7-deficient peritoneal macrophages to mount an appropriate interleukin (IL)-1β response. Comprehensive characterization using a battery of tests assessing locomotion, anxiety- and depression-related as well as social behaviour revealed differences in locomotor and exploratory behaviours. P2X7 KO mice showed slightly increased locomotor activity and reduced anxiety-related behaviour at baseline. Under conditions of chronic stress exposure, genotype-dependent differences largely dissolved while P2X7 deficiency promoted enhanced stress resilience with regard to social behaviour. Taken together, our findings add further evidence for an involvement of the P2X7 in shaping different behavioural responses and their modulation by stressful environments. This novel loss-of-function model will contribute to a better understanding of P2X7 in stress-associated behaviours in basic and translational neuropsychiatric research.

**Supplementary Information:**

The online version contains supplementary material available at 10.1007/s11302-025-10074-x.

## Introduction

Depression is a stress-associated disease and is considered the main cause of disability world-wide [1, 2]. In recent years, aetiopathological concepts increasingly focus on the immune system and its dysregulation as a pertinent factor in depression genesis [3–5]. Among the different mechanisms of interest, purinergic signalling via the ATP-gated P2X7 receptor has been considered particularly promising [6–11] since the receptor is primarily active in chronic inflammatory conditions. This is due to its high activation threshold (EC_50_ for ATP: human P2X7 = 541 μM, rat P2X7 = 130 μM, mouse P2X7 = 2400 μM) [12–14], evoked K^+^ efflux and Ca^2+^/Na^+^ influx [15, 16], as well as its capacity to form non-selective pores for larger organic cations (900 Da) [17–19]. In the central nervous system (CNS), P2X7 is mainly expressed by microglia, oligodendrocytes and astrocytes [12, 19–21]. Neuronal expression remains a matter of debate in the field despite significant progress with regard to novel antibodies and transgenic reporter mice [22–24]. Bacterial artificial chromosome (BAC) transgenic reporter mice have been instrumental for the evaluation of P2X7 expression in different cell types of the brain. However, these tools require careful evaluation prior to their application as demonstrated for the BAC transgenic mice generated by the Gene Expression Nervous System Atlas (GENSAT) project [25]. These mice (*Tg(P2rx7-EGFP)FY174Gsat/Mmucd*) express a soluble EGFP (sEGFP) driven by the *P2rx7* promoter. This mouse line has repeatedly been used as reporter for P2X7 expression under physiological and pathological conditions [26–29]. Only recently, it has been revealed that the sEGFP expression pattern deviates significantly from endogenous P2X7 expression. Moreover, it also ectopically overexpresses P2X7 and co-overexpresses P2X4 as the latter is co-localized on the BAC used for transgenesis [25]. The second BAC transgenic line (Tg(RP24-114E20P2X7-StrepHis-EGFP)Ani) expressing a P2X7 N-terminally fused to EGFP (P2X7-EGFP), faithfully recapitulates endogenous P2X7 expression, e.g. in glial cell populations in the CNS. Still, no expression of the P2X7-EGFP reporter has been detected in neurons [25, 30]. Whether the absent neuronal P2X7 expression is related to the BAC transgenesis, which is associated with a certain degree of variability when comparing different founder lines, or whether it is related to the sensitivity of detection methods remains an open question [30]. Convincing evidence for neuronal expression has been provided on the mRNA level. For instance, mRNA expression in the hippocampal CA3 region is prominent (compare Allen Brain Atlas https://mouse.brain-map.org/experiment/show/75551477) and can be deleted in conditional humanized P2X7 mice when bred to the forebrain glutamatergic neuron-specific Nex-Cre driver line [21]. Moreover, single-cell approaches have confirmed *P2rx7* expression in neurons: the relative mRNA expression in CA3 excitatory neurons (0.0460) has been reported to be in a similar range as the one reported in microglia (0.066) [31].

Upon immune stimulation, cellular trauma and following acute as well as chronic psychosocial stress, ATP is released into the extracellular space [32–34]. Here, it acts as a strong molecular distress signal as part of damage-associated molecular patterns [35]. Aptly, studies have shown that P2X7 signalling is involved in translating environmental adversity, such as acute and chronic psychosocial stress, into inflammatory states associated with depression. This is facilitated by stress-induced ATP release in the CNS, resulting in P2X7-dependent activation of intracellular pathways such as nuclear factor ‘kappa-light-chain-enhancer’ of activated B-cells (NFƙB) and the NLR family pyrin domain containing 3 (NLRP3) inflammasome [4, 12, 16, 33, 34, 36–39]. This jointly leads to IL-1β, IL-18, IL-6 and TNF-alpha as well as amplified ATP and glutamate release, immune activation and neuroplasticity reduction, which ultimately feeds into selected biobehavioural depression features [16, 34]. This aligns with studies in humans and mice that have demonstrated a link between P2X7-mediated immune cell activation, cytokine release and depressive-like behaviours, respectively [3, 7, 16, 33, 34, 40–42].

The association of single-nucleotide polymorphisms (SNPs) in the P2RX7 gene with several neuropsychiatric diseases has repeatedly been reported. In particular, the non-synonymous SNP rs2230912 (1405A > G) which leads to a glutamine by arginine substitution at position 460 (Gln460Arg) in the receptor’s intracellular C-terminal domain has been associated with bipolar disorder and depression [20, 43–45]. Surprisingly, when introduced into the wild-type P2X7, the Gln460Arg mutation alone does not significantly alter its function [46–48]. However, the Gln460Arg polymorphism is part of the haplotype P2X7-4 [49], also designated as H14/H15 [50], and thus is in linkage disequilibrium with the non-synonymous SNP rs1718119 (1068A > G) which confers an alanine to threonine substitution at position 348 (Ala348Thr) in the second transmembrane domain. The Ala348Thr is a gain-of-function mutation and thus has been speculated to be of pathophysiological significance giving rise to increased pro-inflammatory cytokines or neurotransmitters/-modulators to promote disease. Other frequently co-inherited gain-of-function mutations (His155Tyr, His270Arg) might also play a role in this context [49, 51]. Another haplotype-independent observation is that the isolated Gln460Arg mutation is able to abolish receptor function when it is co-expressed together with its wild-type counterpart forming heterotrimers [46, 47]. Accordingly, heterozygous humanized mice harbouring the human wild-type and Gln460Arg P2X7 variant exhibited signs of a prodromal disease state as indicated by reduced sleep quality and an increased susceptibility to chronic stress compared to their homozygous littermates [47].

But even though the functional and genetic link between P2X7 and depression is overall compelling, further research is needed to unravel and specify the role of P2X7 and its polymorphisms on a group and individual level, focusing on gene × environment interactions [45]. In humans, the full-length *P2RX7-A* is accompanied by 9 isoforms (*P2RX7-B-J*) caused by alternative splicing of the *P2RX7* gene. In rodents, 5 splice variants have been described (*P2rx7-a*, *P2rx7-b*, *P2r7-c*, *P2rx7-d* and *P2rx7-k*) [12, 20].

To date, four constitutive P2X7 knockout (KO) mouse lines have been generated (for more detailed reviews see: [44, 52]). Unfortunately, several of these mouse lines have proven to be incomplete KOs since specific splice variants have been demonstrated to escape inactivation [53–55] while in other lines the aspect of splicing has not been investigated. The first published P2X7 KO mouse line generated by GalxoSmithKline (GSK) targeting exon 1 was shown to retain expression of splice variant *P2rx7-k* [54] which is potentially driving the increased P2X7 receptor activity observed in T cells of GSK KO mice [53]. The P2X7 KO line generated by Pfizer carries a selection cassette in exon 13. This mouse line continues to express the C-terminally truncated variants *P2rx7-b* and *P2rx7-c*. In addition, the gene targeting event entails the formation of a novel hybrid transcript which translates into a full-length P2X7 with an alternative C-terminus composed of 75 amino acids encoded by exon 13 and 22 additional P2X7-unrelated amino acids originating from the targeting vector. Another P2X7 KO line, independently generated by Lexicon Genetics, has not been analysed with regards to P2X7 splice variants, but the applied targeting strategy eliminating exon 2 makes it likely that none of the known splice variants will escape inactivation [56]. A similar strategy was applied in a CRISPR/Cas9-mediated approach which, according to the authors, was directed against *P2rx7* exon 2 resulting in deletion of 182 bp between the two guide RNA target sites. However, the two specified target sequences comprise only 93 bp within exon 2 and unfortunately no further details were provided with regards to the exact gene editing event, potential loss of splice variants and its immediate impact on P2X7 receptor function [57]. The general efficiency of targeting exon 2 was demonstrated in KO mice derived from a conditional humanized *P2rx7* allele in which deletion of the second exon also abolished all known mouse transcripts [21]. Humanized conditional P2X7 KO mice have already demonstrated their potential to address receptor function in a spatially and temporally controlled manner [21]. Accordingly, another conditional *P2rx7* allele, which has been made available by the European Mouse Mutagenesis (EUCOMM) program, has attracted increasing interest to address P2X7 receptor function with increasing precision [30, 58, 59].

As detailed above, previous findings from available constitutive P2X7 KO mice have to be taken with some caution as they may reflect only the consequences of a partial loss of P2X7 receptor function. To overcome these uncertainties of available constitutive models, we have generated and validated a novel complete P2X7 KO mouse line and assessed anxiety- and depression-related behaviours at baseline as well as following chronic psychosocial stress exposure.

## Materials and methods

### Animals

All animal experiments were conducted with the approval of and in accordance with the Guide of the Care and Use of Laboratory Animals of the Government of Upper Bavaria, Germany. Mice were group-housed under standard lab conditions (22 ± 1 °C, 55 ± 5% humidity) and maintained under a 12-h light–dark cycle with food and water ad libitum. All experiments were conducted with adult male mice (age: 2–5 months).

### Generation of P2X7 KO mice

**Fig. 1:**
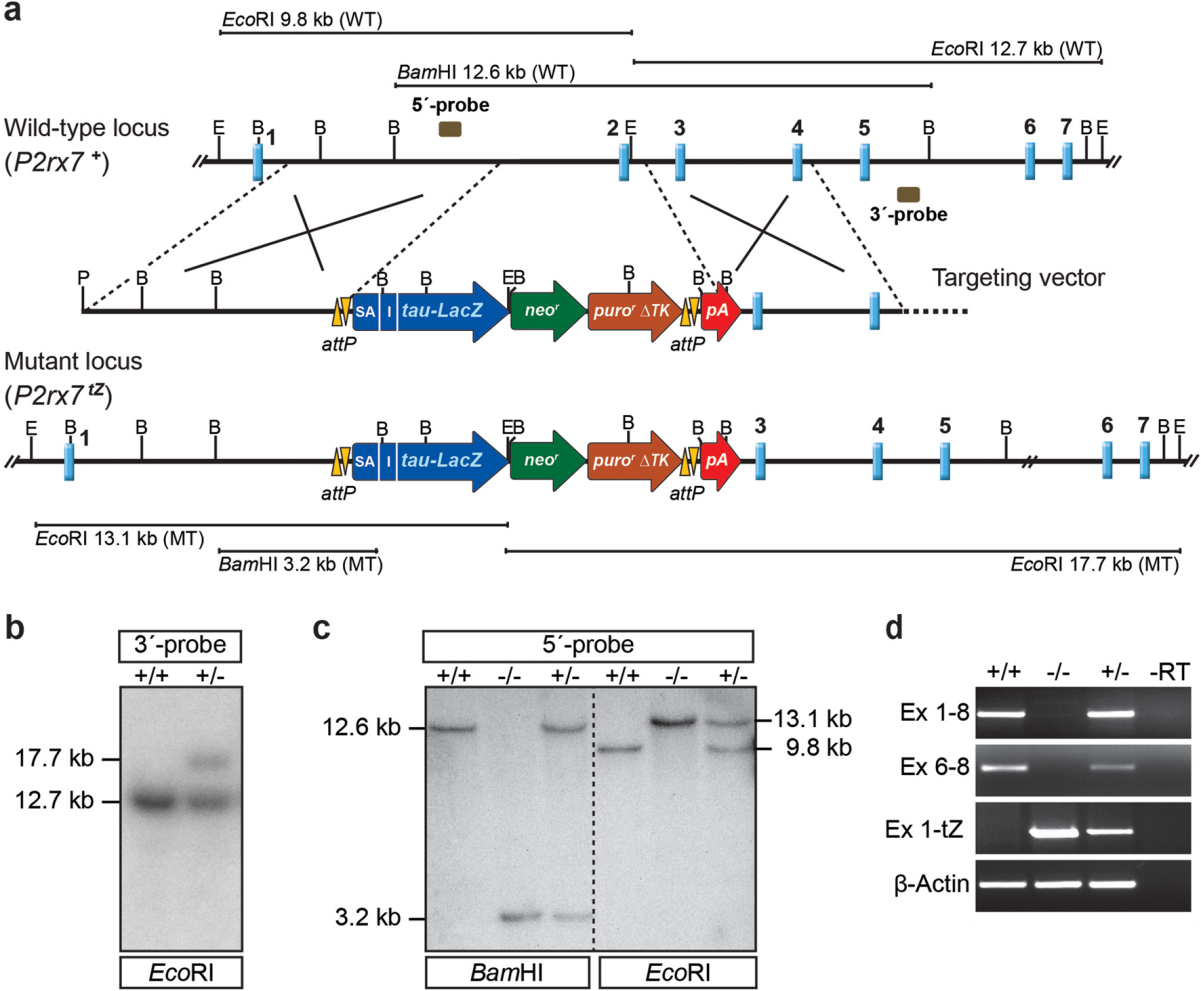
Generation of P2X7 knockout mice. **a** Targeting strategy with partial restriction maps of the wild-type *P2rx7* locus, targeting vector and mutant locus following homologous recombination. **b** Southern blot analysis of wild-type and targeted ES cell clones. The 3′-probe was hybridized to *Eco*RI-digested genomic ES cell DNA. The targeted allele is indicated by the presence of an additional 17.7-kb fragment. **c** Southern blot analysis of F_2_ mice. The 5′-probe was hybridized to *Bam*HI and *Eco*RI-digested genomic DNA. The targeted allele is indicated by the presence of a 3.2-kb *Bam*HI fragment and a 13.1-kb *Eco*RI fragment, respectively. **d** RT-PCR analysis using brain-derived cDNA as template, demonstrates the replacement of *P2rx7* wild-type mRNA by a fusion transcript of exon 1 with the *tau-LacZ* reporter. Abbreviations: B, *Bam*HI; E, *Eco*RI; Ex, Exon; I, internal-ribosomal entry site; pA, polyadenylation signal; SA, splice acceptor

For disruption of the murine *P2rx7* gene, a targeting vector was constructed, which allowed for subsequent recombinase mediated cassette exchange (RMCE) via phiC31 integrase and which contains a *tau-LacZ* reporter gene. Homology arms (HAs) were amplified by polymerase chain reaction (PCR) from genomic DNA of embryonic stem cells (ES) (TBV2, 129S2/SvPas) using Herculase (Stratagene, Heidelberg, Germany) and cloned via the TOPO TA cloning kit (Invitrogen, Karlsruhe, Germany). The 6.0-kb 5′-HA was amplified with primers 5′-PacI-GTC-ATG-TGA-CAA-CTG-CAT-GC-3′ and 5′-MfeI-GCT-GGA-TCA-TCA-GAC-TCT-GT-3′, the 4.0-kb 3′-HA was amplified with primers 5′-AscI-AGT-TTG-CAA-AGC-CGA-GAA-AA-3′ and 5′-EcoRI-GTC-TTT-TTG-CAA-GGC-TGA-GG-3′. Sequencing (Sequiserve, Vaterstetten, Germany) verified the HAs and confirmed that the 5′-HA was inserted into a previously cloned shuttle vector via PacI/MfeI and the 3′-HA via AscI/EcoRI. The HAs enframe a 9.5-kb reporter–selection cassette comprising the following components (from 5′ to 3′): adenovirus type 2 RNase gene splice acceptor (SA), encephalomyocarditis virus internal ribosome entry site (IRES), *tau-LacZ* reporter gene, bovine growth hormone polyadenylation sequence (bGHpA), PGK-Neo-bGHpA, PGK-PuroΔTK-bGHpA and 3 × pA (PGK pA, 2 × SV40 pA). The entire reporter-selection cassette is flanked by RMCE compatible *attP* sites. The vector was designed to substitute 2.3 kb including exon 2 of the *P2rx7* gene that will result in a frameshift and complete loss of P2X7 receptor function (Fig. 1a). The SA-IRES-tau-LacZ-bGHpA and the 3 × pA were isolated and modified from a previously constructed ROSA26 targeting vector [60]. The PGK-PuroΔTK-bGHpA was isolated from pYtC37 [61]. Probes used for identification of homologous recombination events were amplified by PCR from genomic DNA and cloned using the TOPO TA cloning kit. 5′-probe: forward 5′-TAG-TCT-GGC-CCA-AGG-AAC-TG-3′ and reverse 5′-AGT-CCC-TGG-AGC-AAA-CAC-AG-3′ (633-bp); 3′-probe B: forward 5′-AGG-CTA-AGA-TGC-TGG-CAA-TGC-3′ and reverse 5′-CCC-ATG-GAC-ACT-CCT-CAC-AC-3′ (499-bp) (Fig. 1a). The linearized (via *Pac*I) targeting vector bearing 10.0-kb homology to the murine *P2rx7* locus was electroporated into TBV2 (129S2/SvPas background) ES cells. Mutant ES cell clones were identified by Southern blot analysis of genomic ES cell DNA digested with *Eco*RI using the 3′ external probe (Fig. 1b). Mutant ES cells were used to generate chimeric mice by blastocyst injection. Germline transmission of the modified *P2rx7* allele was confirmed in offspring from male chimeras bred to wild-type C57BL/6 J females. Subsequently, mice were maintained on a mixed 129S2/SvPas × C57BL/6 J background. Wild-type (WT) and knockout (KO) littermates derived from heterozygous breeding were used in all experiments. Genotyping was performed by PCR using primers: 5′-GCA-GTC-TCT-CTT-TGC-CTC-GT-3′, 5′-GAC-CGA-AGG-CAA-GAA-CTG-AC-3′ and 5′-GGA-AAG-ACC-GCG-AAG-AGT-TTG-3′. Standard PCR conditions resulted in a 484-bp wild-type and a 317-bp mutant PCR product.

### Reverse transcription (RT)-PCR analysis

Expression of *P2rx7* mRNA in brain and in peripheral tissues was analyzed by RT-PCR. First-strand cDNA synthesis from 1 μg total RNA was performed with SuperScript™ II reverse transcriptase (Invitrogen) according to the manufacturer’s protocol using an oligo (dT) primer. The PCRs were conducted using primers: mP2rx7_E1-8_for 5′-TGC-ACA-TGA-TCG-TCT-TTT-CC-3′, mP2rx7_E1-8_rev 5′-ACC-AGC-TGT-CTA-GGT-TGC-3; mP2rx7_E6-8_for 5′-GCC-GAA-AAC-TTC-ACC-GTA-CT-3′, mP2rx7_E6-8_rev 5′-ACC-AGC-TGT-CTA-GGT-TGC-3′; mP2rx7_E1-tau-lacZ_for 5′-TGC-ACA-TGA-TCG-TCT-TTT-CC-3′, mP2rx7_E1-tau-lacZ_rev 5′-GTT-TTC-CCA-GTC-ACG-ACG-TT-3′; mP2rx7_E1-4_for 5′-CAC-ATG-ATC-GTC-TTT-TCC-TAC-3′, mP2rx7_E1′−4_for 5′-GCC-CGT-GAG-CCA-CTT-ATG-C-3′, mP2rx7_E1-4_rev 5′-GGT-CAG-AAG-AGC-ACT-GTG-C-3′; β-actin_for 5′-ATC-GTG-CGT-GAC-ATC-AAA-GA-3′, β-actin_rev 5′-ACA-TCT-GCT-GGA-AGG-TGG-AC-3′. PCR products were analyzed by agarose gel electrophoresis together with a DNA marker (Smart Ladder, Eurogentec, Brussels, Belgium).

### In situ hybridization

Mice (2–3 months old) were sacrificed by an overdose of isoflurane. The brains were carefully removed and immediately shock frozen on dry ice. Frozen brains were cut on a cryostat in 20-μm-thick sections. The *P2rx7*-specific probe comprises nucleotides 1373–1794 of GenBank accession no. NM_011027. A riboprobe was generated by PCR, labelled and hybridized as described before [62]. The hybridized slides were dipped in autoradiographic emulsion (type NTB2; Eastman Kodak, Rochester, NY, USA), developed after 6 weeks, and counterstained with cresyl violet.

### X-Gal staining

Staining for tau-LacZ report gene activity was performed as previously described [63].

### Western blot

For detection of P2X7 via Western blot, fresh cortex tissue was homogenized, lysed and subsequently analyzed by SDS-PAGE followed by immunoblotting using a polyclonal rabbit antibody directed against the C-terminal domain of P2X7 (Synaptic Systems, Cat no #177,003; 1:1000). A polyclonal rabbit antibody against βactin (Cell SignallingTechnoloct Cat no # 4967; 1:2000) was used as a loading control.

### Interleukin-1β assay

Mice (2–3 months old) were euthanized by an overdose of isoflurane and peritoneal macrophages were collected immediately by lavage of the peritoneal cavity with Dulbecco’s Modified Eagle’s Medium (DMEM) containing 5% of foetal calf serum and penicillin/streptomycin (100 units/ml and 100 μg/ml, respectively). Lavage fluid from 3 to 5 animals was pooled and cells were collected by centrifugation. Cells were re-suspended, counted and 0.5 × 10^6^ cells/well were plated in 24-well plates. The cells were allowed to attach to the well overnight. The next day, 3 μg/ml LPS were added to each well and cells were primed for 2 h. Cells were then challenged for 30 min with 1 mM of 2′,3′-O-(benzoyl-4-benzoyl)-adenosine 5′-triphosphate (BzATP). Finally, supernatants were collected and analysed for IL-1β with an ELISA kit following the manufacturer’s instructions (Endogen, Pierce Technology, Rockford, IL, USA).

### Calcium imaging

Peritoneal macrophages were obtained as described above. Conventional, wide-field fluorescence imaging was performed using a variable scan digital imaging system (TILL Photonics, Martinsried, Germany) attached to an upright microscope (Axioskop, Zeiss, Jena, Germany; 60 × water immersion objective, N.A. 0.90, Olympus Europe, Hamburg, Germany) and a CCD camera as a sensor (Retiga 2000RV, QImaging, Surrey, Canada). Cells were dye-loaded with the Ca^2+^ -selective dye Fluo-4 (Fluo-4-AM, 1 μM; Excitation 485 nm, Emission > 510 nm) by addition to the cultured cells. Macrophages were incubated at 37 °C in a dark incubator for 1 h. For wide-field imaging, background-corrected fluorescence signals were obtained from defined regions of interest after excitation at 485 nm and images were acquired at 2 Hz. After background subtraction, the fluorescence emission was calculated using TillVision software (TILL Photonics, Martinsried, Germany) and data were analyzed off-line using “IGOR Pro”-Software (WaveMetrics, Inc., Lake Oswego, OR). BzATP or KCl (stock concentrations: 100 mM BzATP, 3 mM KCl) were bolus-applied to the bath containing 2 ml HEPES Ringer (140 mM NaCl, 3 mM KCl, 10 mM glucose, 10 mM HEPES, 1 mM CaCl2 and 4.5 mM sucrose, pH 7.35).

### Chronic social defeat stress (CSDS)

The chronic social defeat was performed as previously described [64]. Food and water were provided ad libitum. In brief, wild-type (WT) and KO littermates were subjected to chronic social defeat stress for 21 consecutive days. They were introduced into the home cage of a dominant CD1 for no longer than 5 min and were subsequently defeated. Mice were closely monitored during the procedure to prevent any significant injuries which might result in inflammatory reactions potentially interfering with the intended psychosocial nature of the stressor. Once the test animals showed defeat, they spent 24 h in the same cage as the resident mouse, separated by a perforated partition that enabled sensory but not physical contact. Every day, experimental mice were exposed to a new unfamiliar resident. Control animals were single-housed in their home cages over the course of the experiment. All animals were handled daily, weight and fur status were assessed every 3–4 days. Behavioural testing was conducted during the last week of the CSDS paradigm in the following order: open field test, dark–light box, social avoidance test, forced swim test. All behavioural tests were performed between 08:00 am and 12:00 pm in a room adjacent to the animal housing room. Recording, tracking and scoring of animal behaviours were carried out using the automated video tracking system ANY-maze (ANY-maze; Stoelting Co, Wood Dale, IL, USA). All tests were performed by an experienced, blinded researcher and according to established protocols. At the end of the experiment, adrenal glands and thymus were collected and weighed.

### Open field test (OFT)

The OFT was conducted in an evenly illuminated and box, open at the top (< 15 lx, 50 × 50 × 60 cm). Mice were placed in a corner of the box and left undisturbed for 15 min. Parameters analysed were total distance travelled and total time in the inner zone (3 × 3 inner squares) and outer zone (16 squares along the box walls).

### Elevated plus maze (EPM)

The EPM consisted of a plus-shaped platform, which is elevated 37 cm above the floor. Two opposing open (30 × 5 cm) and closed (30 × 5 × 15 cm) arms were connected by a central zone (5 × 5 cm). Animals were placed in the centre of the apparatus facing the closed arm and left undisturbed for 5 min. Open arm time (OAT) was calculated as a percentage of time in seconds: open arm time (%) = open arm time/(open arm time + closed arm time).

### Dark–Light Box (DLB)

The DLB test was performed in an apparatus consisting of a secure black compartment (< 5 lx, 15 × 20 × 25 cm) and an aversive, brightly illuminated white compartment (700 lx, 30 × 20 × 25 cm). The compartments are connected by a tunnel (4 × 7 × 10 cm), which allows animals to travel unobstructed between the compartments. To start the experiment, mice were placed in the dark compartment and were left unobstructed for 5 min. Entries in the bright zone were counted if the front paws and half of the animal body were inside.

### Forced swim test (FST)

To assess active and passive stress-coping behaviours, mice were placed in a cylindric glass beaker (24 × 12 cm) filled with water (23 ± 1 °C). Behaviour was videotaped for 6 min. Scoring was performed manually for mobility and immobility by a trained observer.

### Social avoidance test (SAT)

The social avoidance test was conducted as previously described [65]. In brief, mice were placed in an OFT arena for 2.5 min containing an empty wire mesh cage on one side, which is called the interaction zone. During the second 2.5 min, animals were confronted with an unfamiliar male CD1 mouse inside the wire mesh cage. Following this, the ratio between the time in the interaction zone in absence and presence of the CD1 mouse was calculated.

### Corticosterone measurements

To determine plasma corticosterone levels, blood draw was performed at baseline (tail cut) and after CSDS (trunk blood decapitation). Concentrations were measured with a radioimmune assay according to the manufacturers protocol (MP Biomedicals Inc.) and as previously described [65].

### Statistical analysis

Data are expressed as mean with standard error of the mean (S.E.M.) or percentage. Statistical analysis was performed using GraphPad Prism (Version 9.3.1). Statistical significance was defined as *p* < 0.05 and is indexed as follows: *p < 0.05, **p < 0.01, ***p < 0.001 and **** *p* < 0.0001 for group comparisons in figures. Analysis was performed by use of one-way, two-way (main factors: genotype, stress) or repeated-measures ANOVA to identify the sample’s source of variation, followed by Sidak’s multiple comparisons. In case of missing data for repeated measures, a mixed-effects model with post hoc correction with Sidak’s multiple comparison test was performed. Outliers were not removed but discussed post analysis to avoid selection bias. For the main ANOVA factors, the F-value is reported.

## Results

### Establishment of a novel constitutive P2X7 knockout mouse line

Classical gene targeting in murine ES cells was used to disrupt the *P2rx7* gene. Homologous recombination resulted in the replacement of a 2.3-kb segment of the murine gene including exon 2 by a *tau-lacZ* (*tZ*) reporter-selection cassette (Fig. 1a, b). The mutant *P2rx7* allele was successfully transmitted through the germline and in the F_2_ generation, homozygous knockout (*P2rx7 *^*tZ/tZ*^, KO) mice were obtained at expected Mendelian frequency. The modification of the *P2rx7* locus in KO and heterozygous (*P2rx7* ^+*/tZ*^) mice was confirmed via Southern blot analysis in comparison to wild-type (*P2rx7* ^+*/*+^, WT) mice (Fig. 1c). The integration of the reporter-selection cassette results in the loss of the wild-type *P2rx7* transcript and expression of a fusion transcript consisting of *P2rx7* exon 1 and the *tau-LacZ* reporter (Fig. 1d). To further examine *tau-LacZ* reporter gene transcription from the *P2rx7* locus, sections of brain, kidney, liver, spleen and submandibular gland of homozygous mutant mice were subjected to X-Gal staining. Surprisingly, no specific staining was detectable suggesting that the *tau-LacZ* reporter is not sensitive enough to detect comparably low *P2rx7* expression levels.

**Fig. 2:**
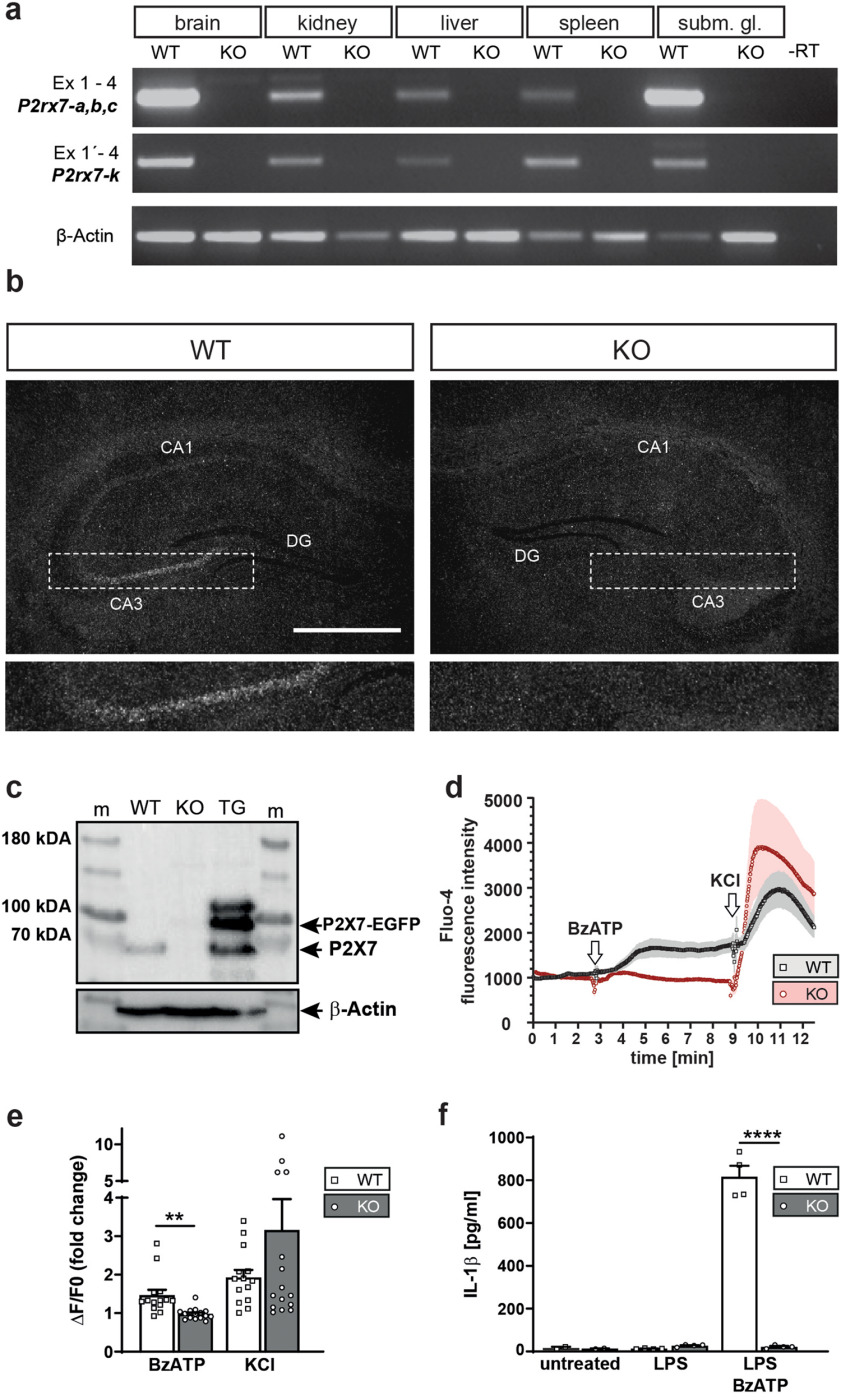
Molecular and functional validation of P2X7 KO mice. **a** Expression analysis of different *P2rx7* splice variants was performed by RT-PCR on mRNA derived from the brain, kidney, liver spleen and submandibular gland (subm. gl.). **b** The loss of *P2rx7* mRNA expression was verified by in situ hybridization using a specific radio-labelled riboprobe detecting endogenous *P2rx7* expression. Depicted are representative dark-field photomicrographs of coronal brain sections of the hippocampus of WT and KO mice. Additionally, a magnification of the hippocampal Cornu ammonis 3 (CA3) region is shown below. Scale bar 500 μm. **c** Western blot demonstrating loss of P2X7 expression in the cortex of KO mice. Tissue from P2X7-EGFP mice (TG) was included as positive control (m, size marker). **d**, **e** Functional assessment of P2X7 receptor activity by calcium imaging. Ca^2+^ influx of peritoneal macrophages of WT and KO mice was measured following BzATP treatment. KCl treatment was used as a positive control. **c** Ca^2+^ data are shown as Fluo-4 fluorescence intensity and as **e** ΔF/F0, where F0 is the resting fluorescence (before stimulation) and ΔF is the peak change in fluorescence from resting levels (WT: n = 14, KO n = 15, unpaired t-test, **p = 0.0012). **f** Functional assessment of P2X7 receptor activity by IL-1β release assay. IL-1β was assessed in peritoneal macrophages of WT and KO mice which were treated with LPS or LPS + BzATP (untreated *n* = 2, treated *n* = 4, one-way ANOVA, *p* < 0.001, *F*_(5,14)_ = 179.5, Sidak’s multiple comparisons test, ****p < 0.0001). Data are presented as mean + standard error of mean (S.E.M.)

To validate the inactivation of the murine *P2rx7* gene in KO mice, its expression was studied on mRNA level by RT-PCR and in situ hybridization. RT-PCR analyses revealed that *P2rx7*-specific splice variants *P2rx7*-*a*, *-b -c* and -*k* were undetectable in the brain and peripheral organs of KO mice (Fig. 2a). Similarly, in situ hybridization using a *P2rx7*-specific riboprobe on brain sections was unable to detect the typical *P2rx7* expression pattern in the hippocampal CA3 area of KO mice (Fig. 2b). Western blot analysis of cortical tissue revealed loss of P2X7 in KO expression on protein level (Fig. 2c).

The successful disruption of the *P2rx7* gene was further confirmed on a functional level. KO peritoneal macrophages showed a significantly reduced Ca^2+^ influx compared to WT cells in response to BzATP stimulation, while the general response of KO and WT macrophages to KCl treatment was comparable (Fig. 2d, e). Moreover, peritoneal macrophages derived from KO mice were unable to mount a normal IL-1β response following combined LPS and BzATP stimulation (Fig. 2f).

Similar to previous reports on constitutive P2X7 deficient mice [21, 56, 66, 67], KO mice exhibited normal development at birth. Also, postnatal development was indistinguishable from that of heterozygous and WT littermates. Inspection of external and internal organs at different ages did not reveal any macroscopic abnormalities in KO mice. No pathological alterations were detected upon histological investigation of various organ systems including brain, heart, lung, liver, spleen, thymus, pancreas, stomach, intestine, ovary, kidney and skeletal muscle (data not shown). Homozygous mutant mice are fertile and reproduce normally.

### Behavioural characterization of the novel P2X7 knockout mouse line

**Fig. 3:**
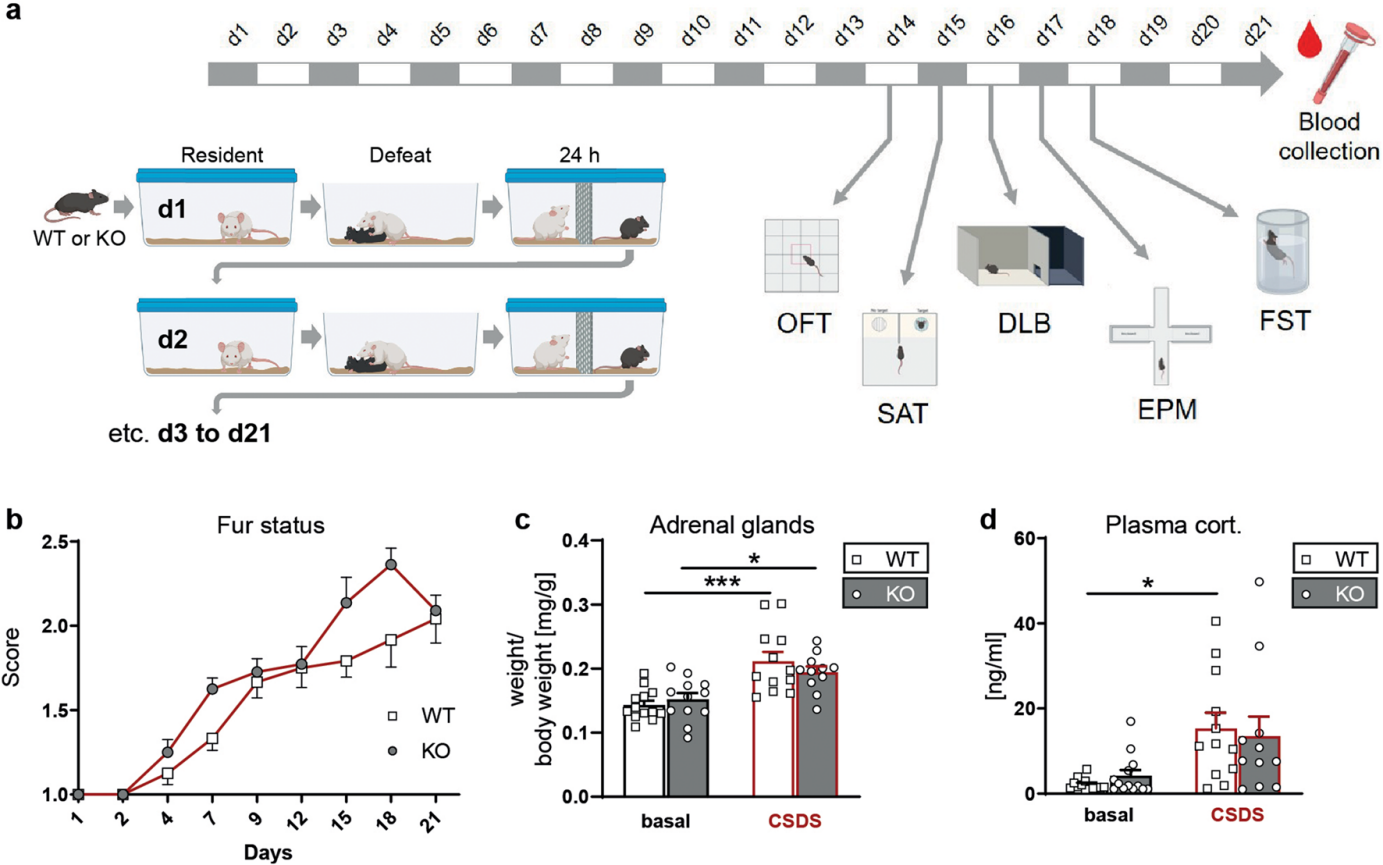
Physiological consequences of chronic stress on P2X7 KO mice. **a** Scheme illustrating the CSDS paradigm and timeline of behavioural tests. Comparison of P2X7 WT and KO mice kept under standard housing conditions (basal) and subjected to CSDS paradigm. **b** Deterioration of the fur status over the course of the CSDS (mixed-effects model, stress: *p* < 0.0001, *F*_(4.717, 100.8)_ = 66,24). Mice kept under basal conditions have a stable sore of 1 (data not shown). **c** Weight of adrenal glands (two-way ANOVA, stress: *p* ≤ 0.0001, *F*_(1,44)_ = 28.34). **d** Morning plasma corticosterone levels (two-way ANOVA, stress: *p* = 0,0003 with *F*_(1,45)_ = 15,71). Data are presented as mean + standard error of mean (S.E.M.), Sidak’s multiple comparisons test, *p < 0.05, ***p < 0.001. *n* = 11–13 animals per group

To examine the behavioural consequences of the P2X7 deficiency, WT and KO mice were subjected to tests assessing locomotion, anxiety-related, social and stress-coping behaviour under baseline (basal) and chronic social defeat stress (CSDS) conditions (Fig. 3a). In the course of three weeks of CSDS the fur status continuously decreased in WT and KO animals (Fig. 3b). In addition, adrenal gland weights at the end of the study were significantly increased in both genotypes as a consequence of the CSDS paradigm (Fig. 3c). Accordingly, morning plasma corticosterone levels were increased in CSDS-treated WT and KO mice compared to mice kept under standard housing conditions (Fig. 3d). Taken together, these physiological measures confirmed the effectiveness of the CSDS paradigm, with similar effects seen in WT and KO animals.

**Fig. 4:**
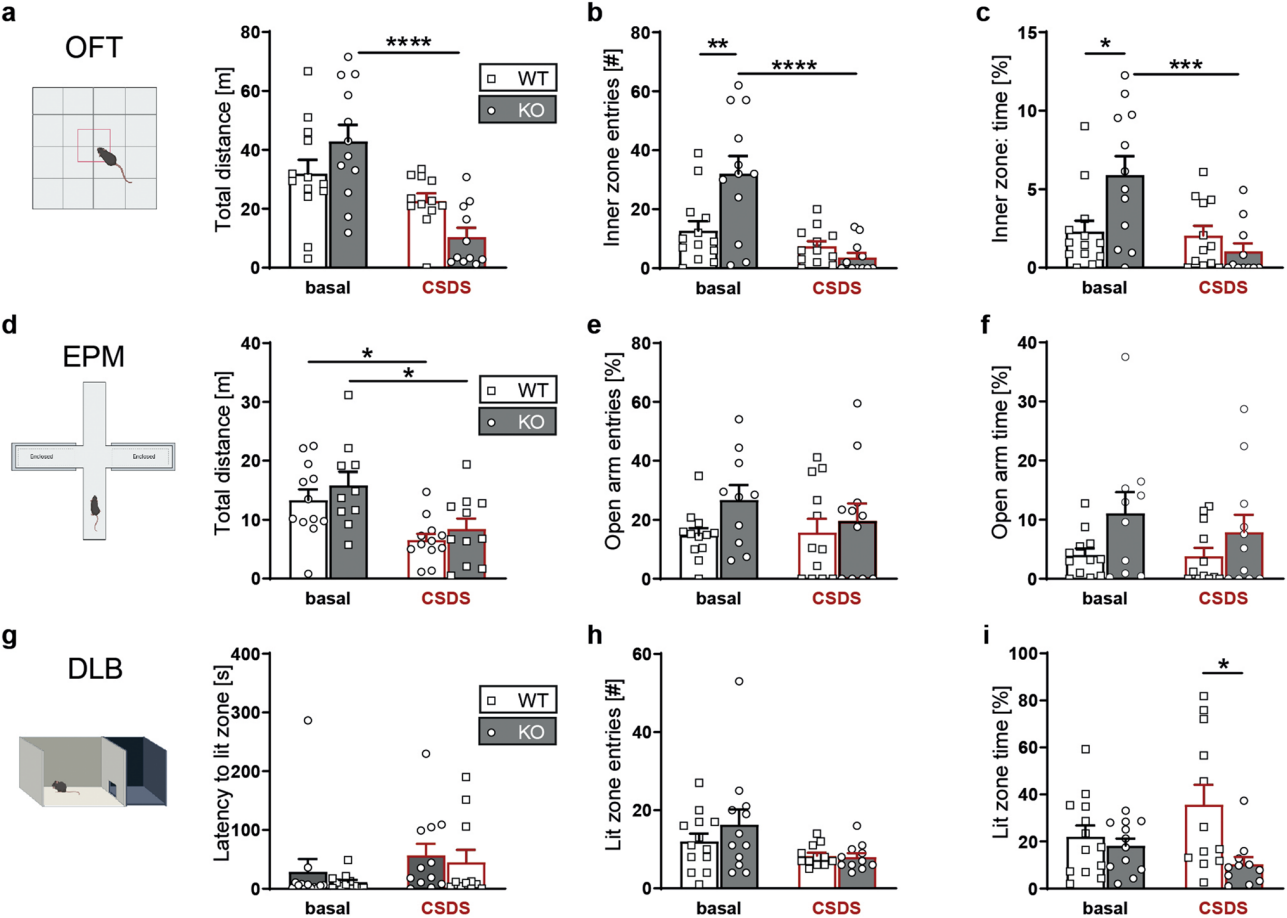
Assessment of locomotor and anxiety-related behaviour in P2X7 KO mice. Comparison of P2X7 WT and KO mice kept under standard housing conditions and subjected to CSDS paradigm. **a–c** Locomotor activity and anxiety-related behaviour were assessed in the open field test (OFT). **a** Distance travelled (two-way ANOVA, stress: *p* < 0.0001, *F*_(1,44)_ = 23.57, genotype × stress: *p* < 0.0098, *F*_(1,44)_ = 7.282), **b** inner zone entries (two-way ANOVA, genotype: *p* = 0.041, *F*_(1,44)_ = 4428; stress: *p* < 0.0001, *F*_(1,44)_ = 20,78; genotype × stress: *p* < 0.0031, *F*_(1,44)_ = 9.790) and **c** time spent in the inner zone of the OFT (two-way ANOVA, stress: *p* = 0.0032, *F*_(1,44)_ = 9.753; genotype × stress: *p* = 0.0071, *F*_(1,44)_ = 7.961). **d–f** Anxiety-related behaviour was assessed in the elevated plus maze (EPM). **d** Distance travelled (two-way ANOVA, stress: *p* = 0.0002, *F*_(1,41)_ = 16,17), **e** open arm entries and **f** time spent on the open arms (two-way ANOVA, genotype: *p* = 0.0235, *F*_(1, 41)_ = 5,541) of the EPM. **g–i** Anxiety-related behaviour was assessed in the dark–light box (DLB). **g** Latency to enter the lit zone, **h** entries (two-way ANOVA, stress: *p* = 0.0133, *F*_(1,44)_ = 6.662) and **i** time spent in the lit zone (two-way ANOVA, genotype: *p* = 0.009, *F*_(1,44)_ = 7.474) of the DLB. Data are presented as mean + standard error of mean (S.E.M.), Sidak’s multiple comparisons test, *p < 0.05, **p < 0.01, ***p < 0.001, ***p < 0.0001. *n* = 11–13 animals per group

Under baseline conditions WT and KO mice did not differ in their locomotor activity as assessed in the OFT. Chronically stressed mice showed markedly reduced locomotor activity reaching statistical significance in KO animals (Fig. 4a). With regards to anxiety-related parameters, unstressed KO mice showed more entries to the inner zone (Fig. 4b) of the OFT and spent significantly more time in this aversive area compared to their respective controls (Fig. 4c). In addition, CSDS-treated KO mice exhibited a significant reduction of the number of entries as well as of the time spent in the inner zone of the OFT (Fig. 4b, c). This decrease was not observed in WT mice which might be owing the already low measures present under baseline conditions potentially preventing the detection of a further reduction of these readouts (Fig. 4b, c). In the EPM, WT and KO mice showed comparable behaviours with regard to the distance travelled, the entries to and time spent on the open arms (Fig. 4d–f). Similar to the OFT, the CSDS had an effect on the locomotor activity which was significantly reduced in both genotypes (Fig. 4d). In addition to the EPM, we also used the DLB to assess anxiety-related behaviour. Under baseline conditions, WT and KO animals showed no significant difference with regards to the time, entries or latency to enter the aversive lit zone of the DLB (Fig. 4g–i). However, the CSDS uncovered a significant difference between WT and KO mice with regard to the time spent in the lit zone which was significantly reduced in KO compared to WT mice (Fig. 4i). However, neither the latency to enter the lit compartment nor the entries to the aversive compartment of the test apparatus were different between genotypes (Fig. 4g, h).

**Fig. 5:**
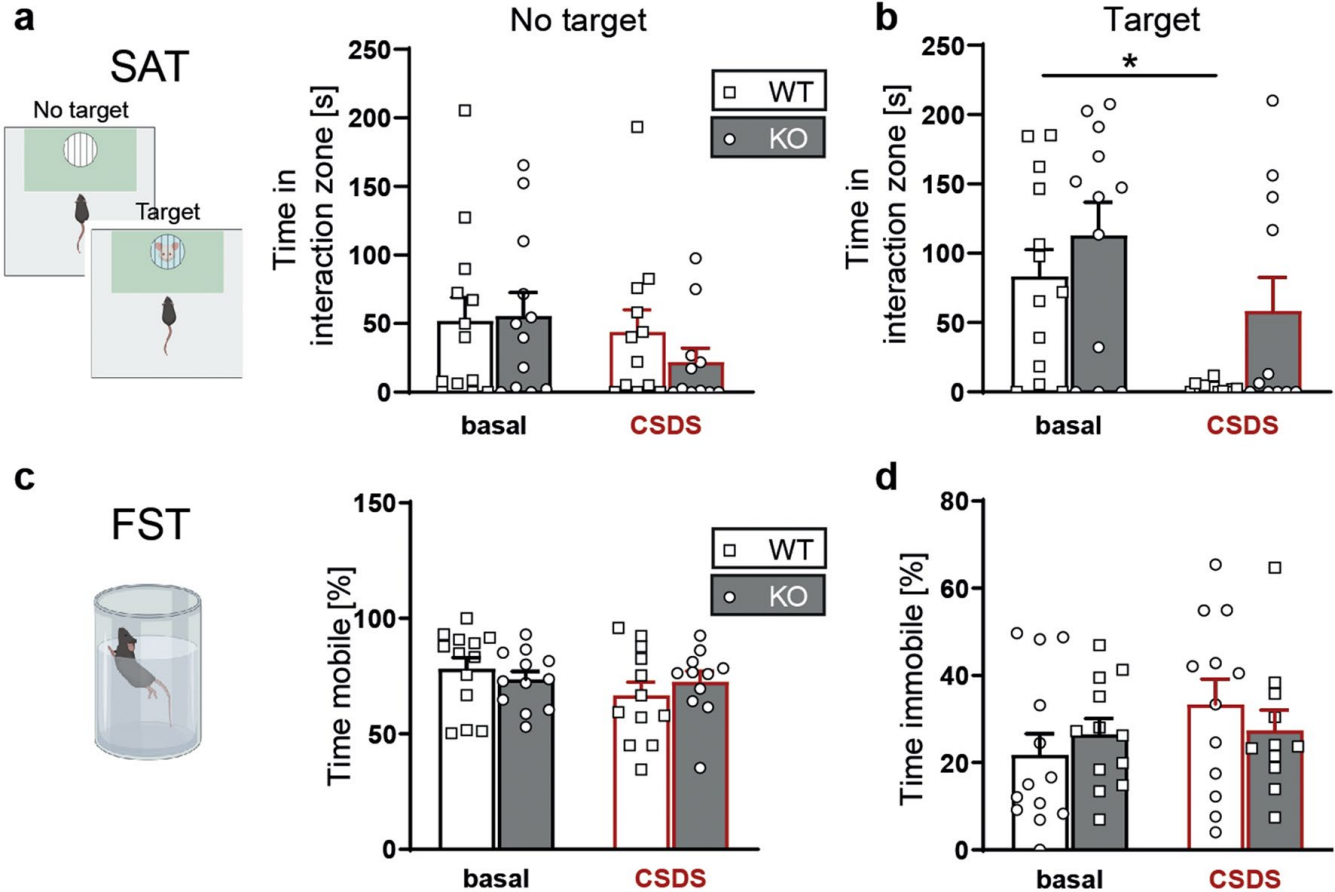
Assessment of social and stress-coping behaviour in P2X7 KO mice. Comparison of P2X7 WT and KO mice kept under standard housing conditions and subjected to CSDS paradigm. **a**, **b** Social avoidance behaviour was assessed in the social avoidance test (SAT). **a** Interaction with empty wire mesh cage (no target) and **b** with the social target (two-way ANOVA, genotype: *p* = 0.02, *F*_(1,36)_ = 5.923; stress: *p* = 0.0047, *F*_(1,36)_ = 9.103; genotype × stress: *p* = 0.0449, *F*_(1,36)_ = 4.32) in the SAT. **c**, **d** Stress-coping behaviour was assessed in the forced swim test (FST). **c** Time spent mobile (active coping) and **d** immobile (passive coping) in the FST. Data are presented as mean + standard error of mean (S.E.M.), Sidak’s multiple comparisons test, *p < 0.05. *n* = 11–13 animals per group

In the SAT, WT and KO mice spent similar time interacting with the empty wire cage and the CSDS had no significant effect on this non-social interaction. In the interaction with the social target no genotype-dependent differences were detectable under baseline conditions (Fig. 5a). However, following CSDS WT mice showed a markedly reduced interaction with the social target which was not observed in KO mice suggesting that the P2X7-deficiency confers resilience to stress-induced social avoidance (Fig. 5b).

Finally, we tested the effect of P2X7 deficiency on stress-coping behaviour in the FST. WT and KO mice that were previously kept under standard housing were indistinguishable with regards to active (mobility) or passive (immobility) coping behaviour. Similarly, the CSDS did not affect coping behaviour in the FST (Fig. 5c, d).

## Discussion

To further unravel the role of P2X7 receptor signaling in basic and translational neuropsychiatry in general and in the context of anxiety and depression research in particular, we generated a novel mouse line in which the known murine splice variants are disrupted and substituted by a fusion transcript comprising *P2rx7* exon 1 which is spliced to a *tau-lacZ* reporter gene. The replacement of exon 2 by a reporter-selection cassette results in the loss of P2X7 receptor mRNA, protein and function as demonstrated via peritoneal macrophages derived from KO mice. Following stimulation by BzATP cells neither showed Ca^2+^ influx nor were they able to elicit an IL-1β response when previously treated with LPS.

Although the fusion transcript was detectable by sensitive RT-PCR, we were not able to visualize P2X7 expression via the *tau-lacZ* reporter gene using X-Gal staining which is presumably related to the comparably low P2X7 expression level. P2X7 mouse mutants generated by GSK [68], Lexicon Genetics [56] and the European Conditional Mouse Mutagenesis (EUCOMM) program also contain a *lacZ* reporter gene. Interestingly, X-Gal staining has only been reported in GSK mice where β-galactosidase activity was exclusively detected in the mandibular gland and in brain ependymal cells [69]. Mice carrying the EUCOMM knockout first allele (*P2rx7*^*tm1a(EUCOMM)Wtsi*^) have been thoroughly screened for reporter gene activity by the International Mouse Phenotyping Consortium (IPMC, (https://www.mousephenotype.org/data/genes/MGI:1339957). From 70 tissues tested, only cells lining the lateral ventricles were identified as *lacZ* positive. We have previously investigated these EUCOMM KO mice using X-Gal staining but could not detect any positive signals (unpublished data). Importantly, the *lacZ* reporter in GSK and EUCOMM mice is integrated at the *P2rx7* start codon or exon 1 is spliced in frame to the reporter gene, respectively. Accordingly, the translation of the reporter mRNA expressed from the *P2rx7* locus will occur in a cap-dependent manner similar to the P2X7 itself. In our KO allele, we instead used an IRES which is known to drive protein expression less efficiently. IRES-dependent gene expression of the second gene in a bicistronic construct is up to tenfold lower than cap-dependent expression of the first gene [70]. Taken together, these findings suggest that the *lacZ* reporter gene is not sufficiently sensitive to detect the comparably low levels of endogenous *P2rx7* expression. These findings are in line with the P2X7-EGFP reporter mouse line which harbours several copies of the reporter construct but also requires antibody-mediated signal amplification for decent visualization of GFP expression [25]. Considering the low and broad expression of the P2X7, the establishment of a Cre recombinase-based reporter approach would provide the remedy to dissect the complexity of P2X7 expression in the brain and periphery with the highest sensitivity [71]. *P2rx7* mRNA expression detected in the pyramidal layer of the hippocampal CA3 region of WT mice is completely lost in KO mice. This finding adds to the growing body of evidence that supports neuronal P2X7 expression and localization in this brain structure [21, 31].

The absence of *P2rx7* splice variants in our novel KO mouse line represents a significant improvement compared to previously generated and widely used constitutive murine P2X7 KO lines from GSK and Pfizer, in which different splice variants escape complete inactivation. This has important implications since changes in alternative splicing patterns, which enable cellular adaption to a changing and challenging environment, are extremely important regulators of physiological and pathological processes [72, 73].

We used our novel constitutive and complete KO mouse line to assess the behavioural consequences of loss of P2X7 receptor function under baseline conditions and following CSDS. We found mild to moderate genotype and genotype × stress effects in most of our behavioural paradigms, while post hoc tests followed by multiple comparison corrections revealed only few statistically significant differences. Moreover, findings were not consistent throughout all employed tests. Locomotor activity in the OFT and EPM, for instance, was found to be higher at baseline in KO compared to WT animals. While in the OFT CSDS induced a pronounced decrease of locomotion in KO animals below WT levels, locomotor activity of KO mice in the EPM remained above WT levels even following chronic stress. A similar phenomenon was observed with regards to anxiety-related readouts in the OFT and in the EPM, which might also be influenced by the alterations in locomotor activity. Though our results are heterogenous concerning the exact effects of P2X7 inactivation on locomotion, its role in locomotor activity is in line with other murine disease models, e.g. for mania or schizophrenia [20, 74]. P2X7-deficient mice displayed reduced social avoidance and more interaction with conspecifics following CSDS. Aptly, chronic stress significantly altered the interaction time in WT but not in KO animals suggesting that the P2X7-deficiency is able to promote stress resilience. These results confirm the importance of P2X7 in regulation of social behaviour, particularly with regard to the translation of chronic psychosocial stress into social withdrawal. Previous rodent studies on P2X7 in neuropsychiatric disorder models of schizophrenia, post-traumatic stress disorder and Rett syndrome, have already supported a role of P2X7 in social behaviour regulation [74–76]. Finally, the FST did not reveal any significant genotype-dependent differences under baseline conditions, which aligns with findings from previous studies [67, 77–79]. However, there are other reports of decreased [56] or even increased [57] immobility in the FST. While CSDS had no effect on FST behaviour, other studies have demonstrated that repeated or prolonged FST protocols resulted in decreased immobility in KO compared to WT mice [67, 78]. Another study using 3 weeks of chronic unpredictable stress (CUS) showed increased immobility in WT mice while the immobility time remained unaffected in KO mice [80]. In a comparable task, the tail suspension test (TST), several studies observed reduced immobility of KO compared to WT mice under baseline conditions (54, 68, 53, 64, 65). A study applying unpredictable chronic mild stress (UCMS) revealed a significant trend towards higher activity in the TST following stress exposure while no baseline difference was detected [81]. The CUS and UCMS paradigms previously applied to P2X7 KO mice differ significantly in their procedures and intensity of stressors applied during the 5-week schedule [80, 81]. However, both paradigms are characterized by the unpredictability and variability of the stressors. These are major differences to the CSDS paradigm. The CSDS involves repeated social defeat and 24-h exposure to a dominant male as a strong psychosocial stressor. Despite the homotypic nature of the stressor, adaptation to the CSDS is rather limited due to daily rotation to a novel unfamiliar dominant male as well as daily variation of the time of day of the defeat procedure. The individual characteristics of the applied stress paradigm are important aspects which have to be carefully considered when interpreting and comparing the behavioural consequences of different stress paradigms [82, 83].

The first behavioural analyses of P2X7 KO mice were conducted with the lines generated by Lexicon Genetics [56, 84] and Pfizer [67, 77, 78]. More recently, additional behavioural studies have been published using KO mice generated by gene editing and based on humanized mice [57, 79]. Comparison of these studies reveals a rather heterogenous and sometimes inconsistent picture with regard to the observed phenotypes in various behavioural assays. To some extent, these discrepancies might reflect environmental confounds, e.g. related to differences in husbandry conditions or exact testing procedures [85] (Table 1). The effect of test laboratory has been identified as one of the key causes for poor replicability in animal research [86]. Moreover, careful inspection of those studies revealed significant differences including the genetic background on which the KO line was generated (Table 1) and the breeding strategy applied for generation of experimental mice ranging from (i) breeding heterozygous mice to obtain WT and KO littermates, (ii) breeding of homozygous WT and KO mice to obtain purely homozygous offspring up to using C57BL/6 mice from a commercial vendor as controls (Table 1). In the latter case, there is no exact specification which C57BL/6 strain was used although significant differences between C56BL/6 substrains (e.g. C57BL/6 J and C57BL/6N) have been repeatedly demonstrated [87]. In the study by Yue and colleagues, information regarding the source of the KO mice is entirely missing [80]. As most KO lines were generated on a 129 background the flanking allele problem may represent another caveat [88], particularly considering that the 129-derived mouse strains express the P2X7-451P variant while C57BL/6-derived lines express the P27R-415 L variant with reduced activity [89]. In addition, the P2X7 expression in CD4^+^ and CD8^+^ T cells is higher in 129- compared C57BL/6-derived mice [90]. Moreover, the sex of the mice as well as the age of the tested animals might influence the behavioural outcome. The effect of age on P2X7-dependent effects was specifically highlighted in the study by Gao and colleagues [57]. The heterogenous behavioural results reported from P2X7 KO mice underscore the importance for accurate reporting of the study’s design but also of the used mouse mutant [85, 91]. The diverse outcome of previous studies renders it difficult to unequivocally disentangle the potential behavioural impact of residual splice variant expression. To minimize environmental and experimental variability, the different P2X7 KO lines would ideally be tested in parallel in a single laboratory.

**Table 1:** Anxiety- and depression-related behaviours reported in constitutive P2X7 KO mice

	**“Reporter-KO”** This study	**“Lexicon”** [56, 84]	**“Pfizer”** [67]	**“Pfizer”** [77, 78]	**“GSK”** [81]	Unknown origin [80]	**“CRISPR/Cas9”** [young mice] [57]	**“Humanized”** [21]
**Allele**	*P2rx7 *^*tm2Jde*^	*P2rx7 *^*tm1Lex*^	*P2rx7 *^*tm1Gab*^	*P2rx7 *^*tm1Gab*^	*P2rx7 *^*tm1Ipch*^	n.s	n.s	*P2rx7 *^*tm1.2Jde*^
**ES cells/zygotes** [genetic background]	129S2/SvPas	129S5/SvEvBrd	129P2/OlaHsd	129P2/OlaHsd	n.s	n.s	C57BL/6	129S2/SvPas
**Sex**	♂	♂	♀	♂	♂	♂ + ♀	♀	♂
**Breeding scheme**	Littermates	Homozygous breeding	n.s	Littermates	Littermates	KO vs C57BL/6	KO vs C57BL/6	Littermates
**Light cycle**	Regular	Regular	Reversed	Regular	Reversed	n.s	n.s	Regular
**TST** [immobility]	n.d	**⇓**	**⇓**	**⇓**	** = **	n.d	n.d	** = **
**FST** [immobility]	** = **	**⇓**	** = **	** = **	n.d	** = **	**⇑**	** = **
**FST, repeated** [immobility]	n.d	n.d	**⇓**	n.d	n.d	n.d	n.d	**⇓**
**FST, longer** [immobility]	n.d	n.d	n.d	**⇓**	n.d	n.d	n.d	n.d
**OFT** [distance]	** = **	** = **	n.d	** = **		** = **	**⇓**	n.d
**OFT** [entries to center]	**⇑**	n.d	n.d	** = **	n.d	n.d	n.d	n.d
**OFT** [time in center]	**⇑**	n.d	n.d	** = **	** = **	n.d	n.d	n.d
**EPM** [entries to OA]	** = **	** = **	**⇓**	** = **	n.d	**⇓**	n.d	n.d
**EPM** [time on OA]	** = **	** = **	**⇓**	** = **	** = **	**⇓**	n.d	n.d
**EPM** [distance]	** = **	n.d	n.d	** = **	n.d	n.d	n.d	n.d
**DLB** [entries lit comp.]	** = **	n.d	** = **	n.d	n.d	n.d	n.d	n.d
**DLB** [time lit comp.]	** = **	n.d	** = **	n.d	n.d	n.d	n.d	n.d
**DLB** [distance]	n.d	n.d	**⇓**	n.d	n.d	n.d	n.d	n.d
**NSFT** [latency]	n.d	** = **	n.d	n.d	n.d	n.d	n.d	n.d
**SPT** [preference]	n.d	n.d	n.d	** = **	n.d	n.d	**⇓**	n.d
**SPT after LPS** [preference]	n.d	n.d	n.d	**⇑**	n.d	n.d	n.d	n.d

Alterations are indicated as change of KO compared to WT. *n.s.*, not specified; *n.d.*, not determined or not reported; *NSFT*, novelty suppressed feeding test; *SPT*, sucrose preference test

Taken together, we have functionally validated and behaviourally characterized a novel P2X7 KO mouse line. Its major novelty lies in the absence of known murine *P2rx7* splice variants. This advantage predestines this mouse line for numerous applications in basic and translational neuropsychiatric research since it allows to address the role of P2X7 in the absence of residual or compensatory expression of splice variants. Even though the observed behavioural effects were moderate, our results provide additional support for the involvement of P2X7 in behavioural control, especially in the regulation of anxiety-related and social behavior. In the future, additional studies involving male and female KO mice will be necessary to deepen our understanding of the genetic, molecular including neuroimmune, and behavioural effects of P2X7 in the context of stress-associated psychiatric disorders.

## Supplementary Information

Below is the link to the electronic supplementary material.

Supplementary file1 (DOCX 1465 KB)

## Data Availability

Data is provided within the manuscript.
